# Normalization of tumor markers and a clear resection margin affect progression-free survival of patients with unresectable pancreatic cancer who have undergone conversion surgery

**DOI:** 10.1186/s12885-023-10529-7

**Published:** 2023-01-14

**Authors:** Xiang Li, Xinyuan Liu, Na Lu, Yiwen Chen, Xiaochen Zhang, Chengxiang Guo, Wenbo Xiao, Xing Xue, Ke Sun, Meng Wang, Shunliang Gao, Yan Shen, Min Zhang, Jian Wu, Risheng Que, Jun Yu, Xueli Bai, Tingbo Liang

**Affiliations:** 1 Department of Hepatobiliary and Pancreatic Surgery, The First Affiliated Hospital, Zhejiang University School of Medicine, Hangzhou, China; 2 Zhejiang Provincial Key Laboratory of Pancreatic Disease, Hangzhou, China; 3 Department of Oncology, The First Affiliated Hospital, Zhejiang University School of Medicine, Hangzhou, China; 4 Department of Radiology, The First Affiliated Hospital, Zhejiang University School of Medicine, Hangzhou, China; 5 Department of Pathology, The First Affiliated Hospital, Zhejiang University School of Medicine, Hangzhou, China; 6 Zhejiang University Cancer Center, Hangzhou, China

**Keywords:** Unresectable pancreatic cancer, Systemic treatment, R0 resection, Tumor marker, Outcome

## Abstract

**Background:**

With the advent of intensive combination regimens, an increasing number of patients with unresectable pancreatic cancer (UPC) have regained the opportunity for surgery. We investigated the clinical benefits and prognostic factors of conversion surgery (CS) in UPC patients.

**Methods:**

We retrospectively enrolled patients with UPC who had received CS following first-line systemic treatment in our center between 2014 to 2022. Treatment response, safety of the surgical procedure and clinicopathological data were collected. We analyzed the prognostic factors for postoperative survival among UPC patients who had CS.

**Results:**

Sixty-seven patients with UPC were enrolled (53 with locally advanced pancreatic cancer (LAPC) and 14 with metastatic pancreatic cancer (MPC)). The duration of preoperative systemic treatment was 4.17 months for LAPC patients and 6.52 months for MPC patients. All patients experienced a partial response (PR) or had stable disease (SD) preoperatively according to imaging. Tumor resection was unsuccessful in four patients and, finally, R0 resection was obtained in 81% of cases. Downstaging was determined pathologically in 87% of cases; four patients achieved a complete pathological response. Median postoperative-progression-free survival (PO-PFS) was 9.77 months and postoperative overall survival (PO-OS) was 31.2 months. Multivariate logistic regression analyses revealed that the resection margin and postoperative changes in levels of tumor markers were significant prognostic factors for PO-PFS. No factors were associated significantly with PO-OS according to multivariate analyses.

**Conclusions:**

CS is a promising strategy for improving the prognosis of UPC patients. The resection margin and postoperative change in levels of tumor markers are the most important prognostic factors for prolonged PFS. Multidisciplinary treatment in high-volume centers is strongly recommended. Prospective studies must be undertaken to resolve the various problems regarding optimal regimens, the duration of treatment, and detailed criteria for CS.

**Supplementary Information:**

The online version contains supplementary material available at 10.1186/s12885-023-10529-7.

## Introduction

Pancreatic cancer (PC) is an extremely malignant tumor type with high lethality in China [1]. In the USA, PC is the fourth leading cause of cancer-related death [2], and has been postulated to be the second leading cause of death within the next decade [3].

For this intractable disease, surgery is considered to the only way to take the tumor under permanent control. However, due to its characteristics of hidden onset, rapid progress and metastasis, ~ 80% of PC patients lose the opportunity to undergo surgery because invasion to vessels or distal metastasis have usually occurred at the time of the initial diagnosis [4].

The treatment plan for PC patients is based mainly on the classification of resectability rather than the stage. According to the recommendation from the National Comprehensive Cancer Network (NCCN), systemic treatment is strongly recommended for patients with locally advanced pancreatic cancer (LAPC) or metastatic pancreatic cancer (MPC).

Recently, reports have shown the advantages of multidisciplinary treatment for transforming unresectable disease to disease eligible for radical surgery, which has resulted in increased resectability and longer survival. FOLFIRINOX (FFX), modified-FOLFIRINOX (mFFX) and gemcitabine plus nab-paclitaxel (GnP) are the main regimens for all stages of PC for patients with good performance status, and enable downstaging before radical surgery [5, 6]. Several studies have shown that patients with LAPC who accept systemic treatment based on first-line regimens can achieve nearly the same outcome as that obtained by patients with initially resectable PC after successful surgery with curative intent [7, 8]. Moreover, the duration of postoperative-overall survival (PO-OS) between patients with LAPC and patients with MPC is not significantly different, which indicates the potential of conducting conversion surgery (CS) for MPC patients [9]. It has been reported that patients with initially unresectable pancreatic cancer (UPC) who undergo CS can achieve PO-OS of 25.1 months [10]. CS for selected UPC patients has become a “hot topic” in PC treatment. However, the safety and efficacy of CS has not been demonstrated in patients with UPC disease.

We evaluated 67 UPC patients who had recieved downstaging surgery following first-line systemic treatment in our center between 2014 to 2022. We wished to discover the postoperative outcome and potential factors strongly associated with survival for LAPC patients and MPC patients.

## Methods

### Study design and patient cohort

In this retrospective study, we enrolled 67 patients with UPC (53 LAPC and 14 MPC) who underwent CS after systemic treatment between April 2014 and March 2022 at our center. The protocol for this retrospective study was approved by the Ethics Committee of The First Affiliated Hospital, Zhejiang University School of Medicine (Hangzhou, China). Patients were diagnosed with PC by histology. LAPC or MPC was verified based on the resectability definition set by the NCCN. LAPC was defined as encasement of the superior mesenteric artery (SMA) or celiac axis (CA) > 180°, abutment of the inferior vena cava, unreconstructable superior mesenteric vein/portal vein (SMV/PV) encasement/occlusion, and aortic invasion/encasement. In addition, all patients were reviewed by a multidisciplinary board for cancer treatment to finalize the treatment strategy, evaluate the response to treatment and make decisions regarding the surgical procedure.

### Regimens of systemic therapy

Some enrolled patients were selected from a prospective clinical study conducted previously in our clinical center. The regimens for these patients strictly followed the standard of clinical research (NCT03977272 and NCT03983057). mFFX (2-weekly schedule) was administered using oxaliplatin (68 mg/m^2^ bodyweight), leucovorin (400 mg/m^2^), irinotecan (135 mg/m^2^) and subsequent intravenous infusion of fluorouracil (2400 mg/m^2^) over 46 h. The GnP regimen consisted of gemcitabine (1000 mg/m^2^) and nab-paclitaxel (125 mg/m^2^) administered on days 1, 8, and 15, every 28 days. Sequential use of GnP and mFFX was set as a 28-day treatment cycle of GnP followed by an additional two doses of mFFX. During each treatment session, patients were treated symptomatically and monitored closely. Adverse effects were recorded according to Common Terminology Criteria for Adverse Events (CTCAE) 4.0. High-resolution computed tomography (CT) of the lungs, contrast-enhanced magnetic resonance imaging (MRI) of the liver and contrast-enhanced CT of the abdomen were undertaken every 2 months to evaluate the responseto treatment.

### CS and adjuvant therapy

A multidisciplinary board in our institution considered surgical exploration for UPC if the following eight eligibility criteria were met: (i) no deterioration of performance status with willingness for surgery from the patient; (ii) radiologic response to systemic treatment according to Response Evaluation Criteria in Solid Tumours (RECIST) criteria; (iii) CR, PR, or SD with the possibility of R0 resection; (iv) improvement of vessel involvement was not essential; (v) marked decrease in cancer antigen (CA)19–9 level in patients with CA19-9 > 200 U/mL at the diagnosis; (vi) metastases were limited to a solitary organ (MPC); (vi) ≤ 3 active metastatic lesions irrespective of their distribution within the liver according to preoperative imaging studies (MPC) and hepatic lesions were technically resectable (MPC); (vii) intensive control of pulmonary metastasis by systemic treatment and suitable for further stereotactic body radiotherapy (SBRT)-mediated local control or resection (MPC). Surgical procedures were selected based on the location of and invasion by the tumor, but vessel resection/reconstruction were considered (if necessary) intraoperatively. Data regarding pathology grade, resection margin, lymph-node invasion and the pathologic response were collected. The College of American Pathologists (CAP) scoring system was employed to evaluate the pathological response to tumor resection [11]: grade 0 (complete response), no remaining viable cancer cells; 1 (moderate response), only a small cluster or single cancer cells remaining; 2 (minimal response), residual cancer remaining, but with predominant fibrosis; 3 (poor response), minimal or no tumor killing with extensive residual cancer. Postoperative status was monitored carefully. Postoperative complications were graded according to the International Study Group on Pancreatic Surgery classification and Clavien–Dindo system. Patients completed imaging examinations and laboratory tests to assess tumor progression within 1–2 months after surgery, and continued to have adjuvant chemotherapy postoperatively.

### Study outcomes and statistical analyses

The primary outcomes were postoperative-progression-free survival (PO-PFS) and PO-OS. We followed up patients by telephone communication every 3 months. Statistical analyses were done with Prism 9 (GraphPad, San Diego, CA, USA) and SPSS 26.0 (IBM, Armonk, NY, USA). Results are given as the median (range) or mean (standard deviation). PFS and OS were calculated using the Kaplan–Meier method and compared using the log‐rank test. Factors with *P* < 0.05 upon univariate analysis without potential confounding were included in multivariate logistic regression analysis. P < 0.05 was considered significant.

## Results

### Patient characteristics

Sixty-seven patients with UPC received systemic treatment and CS between April 2014 and March 2022. The median age of the study cohort was 63 years, and male patients accounted for 61% of cases (Table 1). Pain was the most common clinical symptom (75% of patients), followed by weight loss (42%) and obstructive jaundice (16%). The tumor was located proximal to the pancreas in 39 cases, and distal to the pancreas in 28 cases. The median level of cancer antigen (CA) 19–9 and carcinoembryonic antigen (CEA) in serum was 231.4 U/mL and 3.5 U/mL, respectively. Patients with MPC had higher levels of tumor markers than LAPC patients.

**Table 1:** Basic characteristics of patients with unresectable pancreatic cancer

	UPC *n* = 67	LAPC *n* = 53	MPC *n* = 14
**Age** (years)	63 (35–76)	62.1 (35–76)	60.4 (45–73)
**Sex**			
Male	41 (61)	31 (58)	10 (71)
Female	26 (39)	22 (42)	4 (29)
** Tobacco smoking**	24 (36)	16 (30)	8 (57)
** Alcohol consumption**	28 (42)	22 (42)	6 (43)
**Presenting symptoms**			
Jaundice	11 (16)	9 (17)	2 (14)
Pain	50 (75)	40 (75)	10 (71)
Weight loss	28 (42)	24 (45)	4 (29)
**Comorbidities**			
Hypertension	23 (34)	20 (38)	3 (21)
Diabetes mellitus	19 (28)	17 (32)	2 (14)
**Location of pancreatic tumor**			
Proximal	39 (58)	32 (60)	7 (50)
Distal	28 (42)	21 (40)	7 (50)
**Distant metastasis**			
Lung nodules	3 (4)	-	3 (21)
Hepatic lesion	11 (16)	-	11 (79)
Peritoneal lesion^a^	1 (1)	-	1 (7)
** Biliary stent/PTCD/PTGD**	13 (19)	11 (21)	2 (14)
** SBRT**	11 (16)	9 (17)	2 (14)
**Tumor markers**^**b**^			
CA19-9 (U/mL)	231.4 (0–12,000)	231.4 (0–12,000)	345.75 (2–12,000)
CEA (U/mL)	3.5 (0.7–61.9)	3.1 (0.7–26)	6.60 (2.8–61.9)

*PTCD* percutaneous transhepatic cholangial drainage, *PTGD* percutaneous transhepatic gallbladder drainage, *SBRT* stereotactic body radiation therapy

^a^There was only one patients suffered hepatic and peritoneal lesions at the diagnosis, radiologic response reached CR for peritoneal lesions

^b^The data of tumor markers is shown as the median and range

### Response to and safety of systemic treatments

Sixty patients accepted systemic therapy based on mFFX regimens (Table 2), among which 17 patients had combined treatment with monoclonal antibody against programmed cell death protein-1 (PD-1) and one patient adopted GnP regimens for second-line therapy after chemoresistance. Besides, four patients accepted GnP regimens and three patients accepted sequential use of GnP and mFFX. The median duration of systemic treatment for LAPC patients was 4.17 months. The median duration of preoperative systemic treatment for MPC patients was 6.52 months. According to imaging, 52% of all cases achieved a partial response (PR) to regimens and the objective response rate (ORR) reached 79% in MPC cases (Supplementary Fig. 1). Assessment using levels of the tumor markers CA19-9 or CEA in serum was done. We found that these levels declined to normal after systemic treatment in 36% of patients, declined to > 50% compared with the pre-treatment condition in 33% of patients, and decreased to < 50% or stable in 19% of patients before surgery. The adverse events of systemic treatment were recorded using CTCAE 4.0. Severe treatment-related adverse events were documented in 50% of all patients. Neutropenia was observed in 23 patients (34%). Eleven patients suffered anemia, eight cases suffered thrombocytopenia and three patients had febrile neutropenia.

**Table 2:** Therapeutic characteristics

	UPC *n* = 67	LAPC *n* = 53	MPC *n* = 14
**Chemotherapy regimens**			
based on mFFX	60 (90)	47 (89)	13 (93)
GnP	4 (6)	3 (6)	1 (7)
Sequential use of GnP and mFFX	3 (4)	3 (6)	0 (0)
** Duration of systemic treatment** (months)	4.53 (1.83–14.47)	4.17 (1.83–12.03)	6.52 (2.33–14.47)
**Treatment response**			
PR	35 (52)	24 (45)	11 (79)
SD	32 (48)	29 (55)	3 (21)
**Change in levels of preoperative tumor markers**			
Decrease to normal	24 (36)	17 (32)	7 (50)
Decrease > 50% without normalization	22 (33)	18 (34)	4 (29)
Decrease < 50% or stable	13 (19)	11 (21)	2 (14)
Negative before treatment	8 (12)	7 (13)	1 (7)
**Adverse events (grade)**			
0	6 (9)	2 (4)	4 (29)
1	10 (15)	8 (15)	2 (14)
2	17 (25)	16 (30)	1 (7)
3	29 (43)	24 (45)	5 (36)
4	5 (7)	3 (6)	2 (14)
**Severe adverse events**			
Neutropenia	23(34)	18(34)	5(36)
Anemia	11(16)	7(13)	4(29)
Thrombocytopenia	8(12)	5(9)	3(21)
Febrile neutropenia	3(4)	3(6)	0(0)
Fatigue	2(3)	2(4)	0(0)

*mFFX* modified-FOLFIRINOX, *GnP* gemcitabine plus nab-paclitaxel, *ALT*, alanine aminotransferase, *PR* partial response, *SD* stable disease

### Surgical outcomes and pathological findings

The local tumor could not be removed by resection in four cases. In the other 63 cases, pancreatoduodenectomy (PD) was done in 31 cases, distal pancreatectomy (DP) in 20 patients, distal pancreatectomy with celiac axis resection (DP-CAR) in 11 cases and total pancreatectomy (TP) in one patient. Reconstruction of the PV or SMV was conducted in 23 cases (22 LAPC and one MPC). Five patients with LAPC underwent arterial construction during resection. Lung metastasis and hepatic metastasis were observed in three and 11 patients, respectively. Synchronous metastasectomy was undertaken in nine patients with hepatic lesions and radiologic CR was obtained in an additional two patients. For patients with pulmonary metastasis, a single lesion disappeared in one patient. In another two patients with multiple lesions, most pulmonary metastasis disappeared after systemic treatment, and SBRT was carried out later.

The median duration (in minutes) of PD, DP and DP-CAR was 360, 253, and 270, respectively, whereas TP took 748 min (Table 3). The median volume of blood loss was ~ 200 mL in different operative procedures except for TP (300 mL). R0 resection (1-mm rule) was achieved in > 80% of patients undergoing PD or DP. Only 73% of patients who underwent DP-CAR achieved R0 resection, which was lower than that for the other operative procedures. The median duration of hospital stay was 14, 13 and 15 days for patients who underwent PD, DP or TP, and it was 19 days for patients who underwent DP-CAR.

**Table 3:** Details of conversion surgery classified by procedure

	PD *n* = 31	DP *n* = 20	DP-CAR *n* = 11	TP *n* = 1
**Type of procedure**				
LAPC^a^	25 (81)	13 (65)	10 (91)	1 (100)
MPC	6 (19)	7 (35)	1 (9)	0 (0)
** Duration (min)**	360 (234–593)	253 (133–463)	270 (219–420)	748
** Estimated blood loss (mL)**	200 (50–1300)	200 (100–1300)	200 (100–1000)	300
**Margin (microscopic)**				
R0	25 (81)	17 (85)	8 (73)	1 (100)
R1	4 (13)	2 (10)	2 (18)	0 (0)
R2	2 (6)	1 (5)	1 (9)	0 (0)
**Postoperative duration of hospital stay (days)**	14 (6–53)	13(5–30)	19 (10–33)	15
**Complications**				
Pleural effusion	13 (42)	9 (45)	6 (55)	0(0)
Ascites	13 (42)	8 (40)	2 (18)	0(0)
Chylus fistula	8 (26)	2 (10)	4 (36)	0(0)
Pancreatic fistula	3 (10)	5 (25)	5 (45)	0(0)
Infection	3 (10)	2 (10)	4 (36)	1 (100)
**Clavien**–**Dindo grade**				
0	7 (23)	4 (20)	0 (0)	0 (0)
I	16 (52)	6 (30)	3 (27)	0 (0)
II	4 (13)	4 (20)	4 (36)	0 (0)
III	4 (13)	6 (30)	4 (36)	0 (0)
IV	0 (0)	0 (0)	0 (0)	0 (0)
V	0 (0)	0 (0)	0 (0)	1 (100)
** 30-day unplanned readmission to hospital**	3 (10)	5 (25)	1 (0)	0 (0)
** Postoperative death (within 90 days)**	0 (0)	0 (0)	0 (0)	1 (100)
** Unplanned reoperation**	2 (6)	2 (10)	0 (0)	1 (100)

^a^Four patients continued to have unresectable pancreatic cancer intraoperatively and were excluded

*PD* pancreatoduodenectomy; *DP* distal pancreatectomy; *TP* total pancreatectomy; *DP-CAR* distal pancreatectomy with celiac axis resection

Postoperative complications varied according to which surgical procedure was done. Patients who undwerwent PD mainly suffered from pleural effusion and asicites, both accounting for 42%. Pleural effusion, ascites and pancreatic fistulae were the most prevalent complications of patients who had DP, accounting for 45%, 40% and 25%, respectively. Among the 11 patients who had DP-CAR, the prevalence of pleural effusion, chylus fistula, pancreatic fistula and infection was > 36%. According to the Clavien–Dindo classification, patients who suffered severe complications of grade III and above after DP or DP-CAR accounted for 30% and 36%, respectively. The prevalence of severe complications after PD was 13%. Otherwise, five patients who underwent DP were readmitted to hospital within 30 days, and two patients accepted an unplanned repeat procedure because of hemorrhage and intestinal obstruction. Meanwhile, three patients who had PD were readmitted to hospital within 30 days and two patients accepted an unplanned repeat procedure (laparotomy because of a broken abdominal drainage tube and abdominal hemorrhage). Patients who underwent TP suffered from peritoneal infection and bleeding died within 90 days. The details of CS classified by resectability are demonstrated in Supplementary Table 1.

The mean diameter of 63 resected local tumors was 26.98 mm, and 41% of patients had positive lymph nodes. According to postoperative pathology (Table 4), Tumor-node-metastasis (TNM) downstaging was observed in 87.3% of patients: three patients had stage 0, 15 cases were in stage I, 19 patients had stage II, 16 patients were in stage III and 10 patients had stage IV. With regard to the pathological response based on the CAP grading system, grade 0 was achieved in four patients (6.3%) (Supplementary Fig. 2), grade 1 in 10 patients (15.9%), grade 2 in 41 patients (65.0%) and grade 3 in eight patients (12.7%). Notably, a major pathological response was achieved in 22.2% of cases (14/63).

**Table 4:** Pathology and adjuvant treatment of patients who underwent conversion surgery

	UPC *n* = 63 ^a^	LAPC *n* = 49^a^	MPC *n* = 14
**Local tumor diameter (mm)**	26.98	29.08	19.64
**Number of dissected lymph nodes**	12 (1–47)	12 (1–47)	11 (1–20)
**Positivity of lymph nodes**	26 (41)	22 (45)	4 (29)
**Pathological response based on CAP grade**			
0	4 (6)	1 (2)	3 (21)
1	10 (16)	10 (20)	0 (0)
2	41 (65)	33 (67)	8 (57)
3	8 (13)	5 (10)	3 (21)
**TNM classification (restage)**			
0	3 (5)	1 (2)	2 (14)
I	15 (24)	11 (22)	4 (28)
II	19 (30)	19 (39)	0 (0)
III	16 (25)	16 (33)	0 (0)
IV	10 (16)	2 (4)	8 (57)
**Adjuvant therapy**	56 (89)	42 (86)	14 (100)
**Adjuvant therapy regimen**			
mFFX	38 (60)	27 (55)	11 (79)
GnP	7 (11)	5 (10)	2 (14)
Other	11 (17)	10 (20)	1 (7)

^a^Four patients continued to have unresectable pancreatic cancer intraoperatively and were excluded

*CAP grade* The College of American Pathologists (CAP) scoring system

### Adjuvant chemotherapy and postoperative outcomes

Excluding the one patient who died in the perioperative period, adjuvant chemotherapy was administered in 56 patients, and the remaining six patients did not undergo further treatment, primarily due to physical weakness (Table 4). The most common use of adjuvant treatment was based on mFFX regimens (38 patients), whereas seven patients accepted GnP regimens. Recurrence was observed in 45 patients, which accounted for 73% of the 62 patients who underwent tumor resection. Four patients achieved a pathological complete response (pCR) according to postoperative pathology. Among these four patients, one patient suffered recurrence with a metastatic tumor in the lungs. The recurrence rate of pCR patients were 25%.

**Fig. 1:**
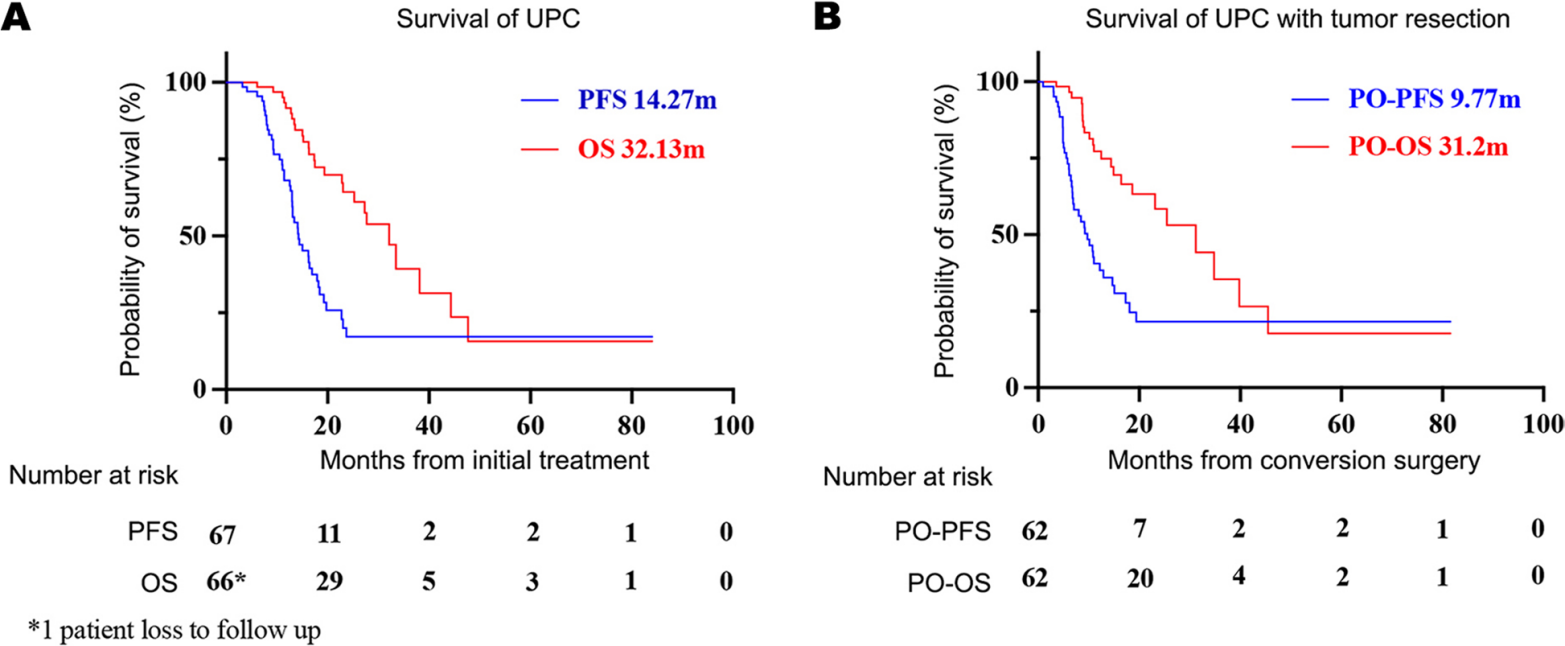
Survival data of UPC patients. **A** OS and PFS of all UPC patients, **B** PO-OS and PO-PFS of UPC patients who underwent tumor resection

**Fig. 2:**
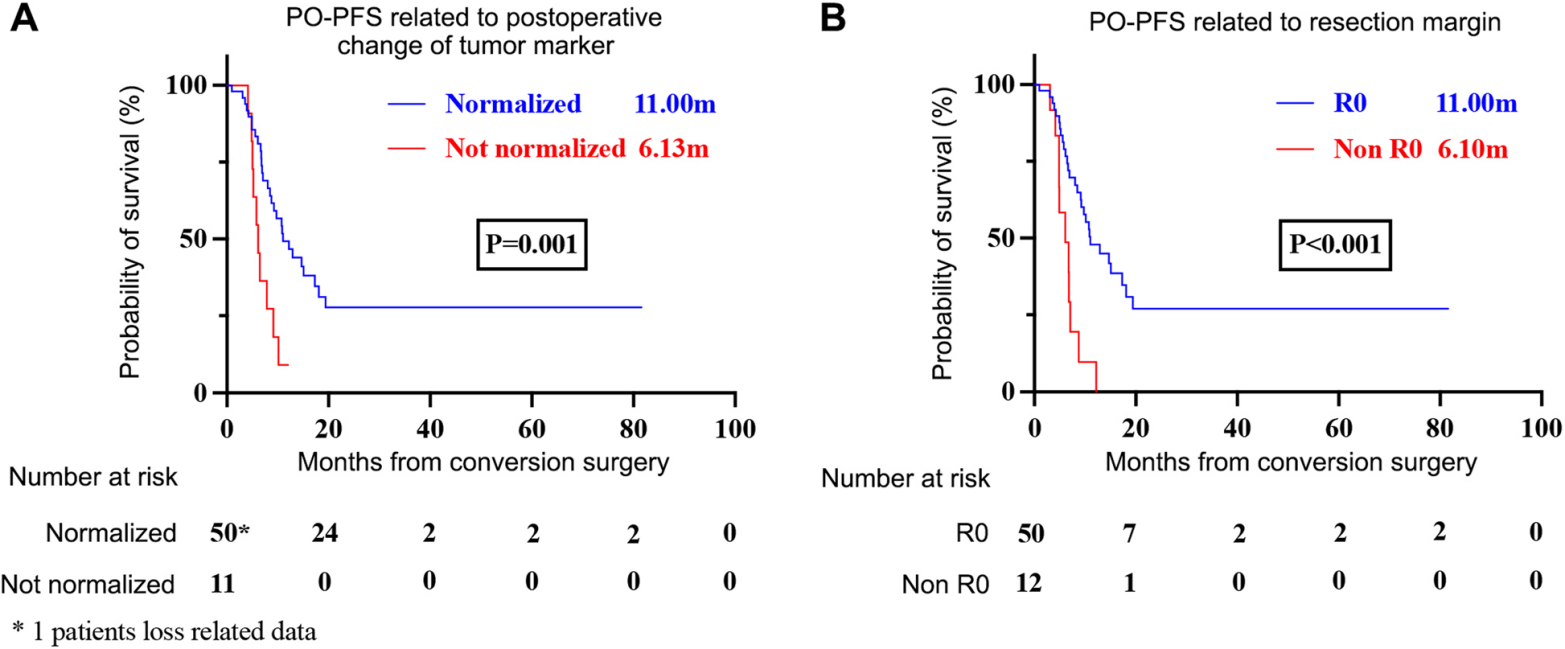
PO-PFS-related prognostic factors. **A** PO-PFS according to the change in the level of tumor markers; **B** PO-PFS depending on the resection margin

Median PFS of the 67 patients was 14.27 months, and median OS was 32.13 months (Fig. 1A). Excluding the four cases who did not undergo tumor resection and the one patient who died in the perioperative period, median PO-PFS was 9.77 months and PO-OS was 31.2 months (Fig. 1B). For LAPC cases, PO-PFS and PO-OS was 10.13 and 31.2 months, respectively (Supplementary Fig. 3A). For the 14 MPC cases, PO-PFS was 7.10 months while OS did not reach (Supplementary Fig. 3B). PO-PFS of patients with lung metastasis was 7.1 months, and 12.2 months of patients with hepatic lesions (*p* = 0.138). Patients with hepatic lesions experienced PO-OS of 25.47 months and PO-OS was not reached in patients with lung lesions. Through univariate and multivariate logistic regression analyses (Table 5), we explored the prognostic factors for PO-PFS and PO-OS among 62 patients who underwent CS successfully. The resection margin was related strongly to PO-PFS (P < 0.001), Response Evaluation Criteria in Solid Tumours (RECIST) evaluation was related to PO-OS (*P* = 0.079), and the postoperative change in the level of tumor markers was related to PFS (*P* = 0.001) and OS (*P* = 0.013) in the univariate analysis (Fig. 2). Lymph-node positivity, CAP score, and change in the level of tumor markers during systemic treatment and TNM staging did not influence PO-PFS or PO-OS. Multivariate logistic regression analyses revealed that the resection margin and postoperative change in the level of tumor markers were significant prognostic factors for PO-PFS (P < 0.001, HR = 0.194, 95%CI: 0.088–0.426; *P* = 0.001, HR = 0.253, 95%CI: 0.114–0.560). No factors were associated markedly with PO-OS according to multivariate analyses.

**Table 5:** Factors influencing postoperative survival

	**Postoperative PFS**				**Postoperative OS**			
	**Univariate analysis**	**Multivariate analysis**			**Univariate analysis**	**Multivariate analysis**		
	**P**	**P**	**HR**	**95%CI**	**P**	**P**	**HR**	**95%CI**
**Age** (> 60 vs. ≤ 60 years)	0.307	-	-	-	0.81	-	-	-
**Sex** (male *vs*. female)	0.280	-	-	-	0.262	-	-	-
**Location of pancreatic tumor** (proximal *vs*. distal)	0.732	-	-	-	0.337	-	-	-
**Tumor diameter** (< 20 *vs*. ≥ 20 mm)	0.165	-	-	-	0.313	-	-	-
**Margin** (R0 *vs*. R1 and R2)	< 0.001	< 0.001	0.194	0.088–0.426	0.102	-	-	-
**LN metastasis** (( +) *vs*. ( −))	0.884	-	-	-	0.058	-	-	-
**RECIST** (PR and CR *vs*. SD)	0.306	-	-	-	0.016	0.079	0.399	0.143–1.114
**Duration of systemic treatment**	0.094	-	-	-	0.08	-	-	-
**TNM staging** (0, I and II *vs*. III and IV)	0.686	-	-	-	0.686	-	-	-
**UPC** (LAPC *vs*. MPC)	0.949	-	-	-	0.414	-	-	-
**Postoperative change in CA19-9/CEA level** (decrease to normal *vs*. not normalized)	0.001	0.001	0.253	0.114–0.560	0.013	0.071	0.405	0.152–1.108
**CAP grading system** (0 and 1 *vs*. 2 and 3)	0.589	-	-	-	0.834	-	-	-

## Discussion

With the advent of intensive regimens such as mFOLFIRINOX and GnP, the ORR has increased considerably compared with that using gemcitabine monotherapy. As a result, the prognosis of PC patients has improved significantly [12, 13]. However, the increase in survival has been only a matter of months.

Surgery is the only treatment to cure PC but it has never been an optimal option for UPC. The clinical benefits of surgery for patients with unresectable disease are controversial [6, 14]. Accompanied with a good response to treatment, some (including UPC) patients regain the opportunity to undergo surgery. Novel combination treatments bring hope to UPC patients and confidence to surgeons. Hence, research on the clinical benefit of CS for UPC has become a hot topic in recent years.

Preoperative systemic treatment can reduce the accuracy of imaging in the evaluation of resectability after treatment owing to treatment-related fibrosis [15, 16]. Moreover, vessel involvement is observed in most UPC patients, and R0 resection is required for combined resection and reconstruction of relevant vessels [17]. Therefore, a lower accuracy of preoperative evaluation and a more invasive procedure may lead to a longer duration of procedure and greater blood loss. However, del Chiaro et al. demonstrated that an aggressive procedure did not result in higher postoperative mortality (2.9% *vs*. 2.6%, *P* = 0.9) or postoperative surgical complications (38.2% *vs*. 25.6%, *P* = 0.2) compared with that using a palliative procedure [17]. Rangelova et al. reported a prevalence of total surgical postoperative complications of 48% in patients who had CS, and 90-day mortality reached 6% [18]. The prevalence of severe complications and postoperative death was 24% and 2%, respectively, in our study, which was relatively low. DP and DP-CAR was undertaken in 49% of patients in our study. Until recently, development of DP-CAR significantly increased the CS rate for tumors of the body or tail of the pancreas that involved the CA. In high-volume centers, the overall prevalence of complications after DP-CAR has been reported to be 42.6%, and only 18.5% of patients developed complications of grade III or worse [19]. Therefore, DP-CAR is accepted widely to be a safe and feasible procedure for UPC patients with downstaging treatment [20]. In our study, 14% of MPC patients developed postoperative complications of grade III or worse, which was significantly lower than that for LAPC cases (26%). One of the most important reasons is that the local tumor in 86% of MPC patients was resectable or borderline-resectable. A pancreatic fistula was one of the most common postoperative complications, and a pancreatic fistula was observed in 13 cases, with one patient dying due to pancreatic fistula-related hemorrhage. Taken together, these data suggest that CS is safe for carefully selected UPC patients in high-volume centers.

Several reports have affirmed the survival benefit of CS for UPC patients [6]. In the present study, the long-term outcomes of UPC patients who underwent tumor resection after systemic treatment were encouraging. Median OS of 32.1 months and median PO-OS of 31.2 months were documented for UPC patients, which were similar to the outcomes for patients with resectable disease. Recently, some scholars have reported on resection of local pancreatic tumors with synchronous metastases. A review by Sakaguchi et al. focused on the usefulness of surgery in MPC patients. They looked at studies involving 428 patients who underwent resection for liver metastases, lung metastases and peritoneal dissemination. Median OS in patients with liver metastases was > 30 months [21]. Surgery for MPC remains controversial. Four criteria of CS for MPC patients are important: (i) a few metastases or occult metastases which were not diagnosed in the preoperative evaluation; (ii) no evidence of multiple-organ disease; (iii) a high probability of obtaining radical excision with an acceptable surgical risk; (iv) good physical status of the patient [22]. In the present study, for 14 MPC cases, PO-PFS was 7.10 months while OS did not reach, which implied that “super-responders” with metastastic disease could also be surgical candidates.

CS was carried out only for an extremely select group of patients who were super-responders to systemic treatment. An important indication for surgery in UPC patients is that radical excision of the tumor can be achieved. Imaging is the most widely used method to evaluate resectability, but the accuracy of imaging decreases markedly after systemic treatment. White et al. demonstrated that restaging unresectability based on CT findings deprived ~ 20% of patients the opportunity for curative resection [22]. Hence, patients without progressive disease who undergo aggressive systemic treatment should be considered as CS candidates. All patients in our study achieved a PR or SD and, finally, a high prevalence of a negative margin of 81% was achieved. Moreover, patients with a PR showed longer PO-OS than patients with SD in the univariate analysis. Our findings are similar to the results documented by Takano and colleagues: early tumor shrinkage during systemic treatment was the only independent prognostic factor for patients with unresectable LAPC who had CS [23]. Positron emission tomography/computed tomography (PET/CT) can be employed to identify the metabolic rate of a tumor before and after treatment, which aids assessment of the response to systemic treatment [24]. It has been reported that a higher post-treatment peak standardized uptake value corrected for lean body mass (SULpeak) and positive metabolic tumor volume/total lesion glycolysis (MTV/TLG) can be used to predict an unfavorable pathological response in patients who have undergone systemic treatment. Therefore, PET/CT could further improve the accuracy of the response evaluation after systemic treatment [25].

The clinical response must also be taken into consideration. Changes in tumor marker levels likely represent a tumor-specific response to systemic treatment. Tsai et al. found that patients with normal preoperative or postoperative CA19-9 levels experienced a doubling in OS compared with patients who did not have normal levels [26]. Similar results were reported by Truty and colleagues: patients with an increased CA19-9 level that was normalized after treatment experienced much longer recurrence-free survival (RFS; 32.8 months) and OS (72.1 months) than that of patients who had an increased CA19-9 level post-treatment (RFS = 11.2 months and OS = 38.4 months) [27]. PFS of patients with a normalized postoperative tumor marker was extended significantly compared with that of patients who did not have a normalized tumor marker level (11.0 *vs*. 6.13 months, P < 0.05) in our study.

The pathological response is the “gold standard” for assessing tumor degeneration/necrosis, and is a prognostic factor. Chatterjee et al. postulated that the major pathological response (CAP grade = 0–1) in tumor specimens was strongly correlated with longer survival in PC patients [28]. In our study, four patients achieved pathological complete remission (CAP grade = 0) and 10 patients had minimal residual tumors (CAP grade = 1). However, there was no significant difference in PO-PFS or PO-OS between patients who achieved a major pathological response and those who did not. Mataki et al. reported no significant differences in survival between patients with different pathological responses, which is similar to our findings [29]. A pCR is defined as the absence of any observable cancer cells on final pathology, but disease recurrence is observed [28]. Blair et al. reported a recurrence prevalence of 46.7% in patients with a pCR [30]. Among the four patients with a pCR in our study, one suffered tumor recurrence to the lungs. The recurrence prevalence of patients with a pCR was 25%.

Our study had three main limitations. First, there was a selection bias for patients. Second, the cohort size was small. Third, international consensus on CS for UPC is not available, so when and how to carry out a surgical procedure is controversial. CS may be the most effective way to lengthen survival in UPC patients, and systemic treatment is the best way to select surgical candidates at this stage. CS prevalence varies among regimens and treatment protocols, but GnP and mFFX are recommended. Several types of systemic treatment, including chemo-immunotherapy, have been applied recently, but the role of immunotherapy is not clear. The usefulness of adjuvant therapy in patients who have completed preoperative systemic treatment and radical excision is not clear.

## Supplementary Information

**Additional file 1:** **Supplementary Figures 1-Figure 3.**

**Additional file 2:** **Supplementary Table 1.**

**Additional file 3:** **Supplementary Table 2.**

**Additional file 4:** **Supplementary Table 3.**

## Data Availability

The datasets generated and analyzed during the current study are not publicly available due to protection the privacy of patients' health information but are available from the corresponding author on reasonable request.

## Abbreviations

UPC: Unresectable pancreatic cancer
CS: Conversion surgery
LAPC: Locally advanced pancreatic cancer
MPC: Metastatic pancreatic cancer
PR: Partial response
SD: Stable disease
PFS: Progression-free survival
OS: Overall survival
RFS: Recurrence-free survival
PO-PFS: Postoperative-progression-free survival
PO-OS: Postoperative overall survival
PC: Pancreatic cancer
NCCN: National Comprehensive Cancer Network
FFX: FOLFIRINOX
mFFX: Modified-FOLFIRINOX
GnP: Gemcitabine plus nab-paclitaxel
SMA: Superior mesenteric artery
CA: Celiac axis
SMV/PV: Superior mesenteric vein/portal vein
CTCAE: Common Terminology Criteria for Adverse Events
CT: Computed tomography
MRI: Magnetic resonance imaging
PET: Positron emission tomography
SULpeak: Peak standardized uptake value corrected for lean body mass
MTV: Metabolic tumor volume
TLG: Total lesion glycolysis
SBRT: Stereotactic body radiotherapy
CAP: College of American Pathologists
CA: Cancer antigen
CEA: Carcinoembryonic antigen
ORR: Objective response rate
PD: Pancreatoduodenectomy
DP: Distal pancreatectomy
DP-CAR: Distal pancreatectomy with celiac axis resection
TP: Total pancreatectomy
pCR: Pathological complete response
