# Supplementary material for: Normalization of tumor markers and a clear resection margin affect progression-free survival of patients with unresectable pancreatic cancer who have undergone conversion surgery
Source: BMC Cancer. 2023 Jan 14;23:49. doi: 10.1186/s12885-023-10529-7 (PMC9840266; doi:10.1186/s12885-023-10529-7)
Supplement: Supplementary file 1 — Additional file 1: Supplementary Figures 1-Figure 3. [file 12885_2023_10529_MOESM1_ESM.zip › Supplementary Figure Legend.docx]

Supplementary Figure 1. Treatment response of representative UPC cases.

1. Pretreatment (*left*) and preoperative (*right*) contrast-enhanced CT of the abdomen of an LAPC patient who had conversion surgery; B) pretreatment (*left*) and preoperative (*right*) contrast-enhanced CT of the abdomen/contrast-enhanced MRI of the liver of a MPC patient who had conversion surgery.

Supplemetary Figure 2. Details of patients with a pathological complete response (pCR).

Detailed information of clinical events for four patients who experienced a pCR during treatment.

Supplementary Figure 3. Survival data of LAPC patients and MPC patients.

A) OS and PFS of all LAPC patients (*left*) and PO-OS and PO-PFS of LAPC patients who had tumor resection (*right*); B) OS and PFS of all MPC patients (*left*) and PO-OS and PO-PFS of MPC patients who underwent tumor resection (*right*).
