# Supplementary figures and images for: Normalization of tumor markers and a clear resection margin affect progression-free survival of patients with unresectable pancreatic cancer who have undergone conversion surgery
Source: BMC Cancer. 2023 Jan 14;23:49. doi: 10.1186/s12885-023-10529-7 (PMC9840266; doi:10.1186/s12885-023-10529-7)

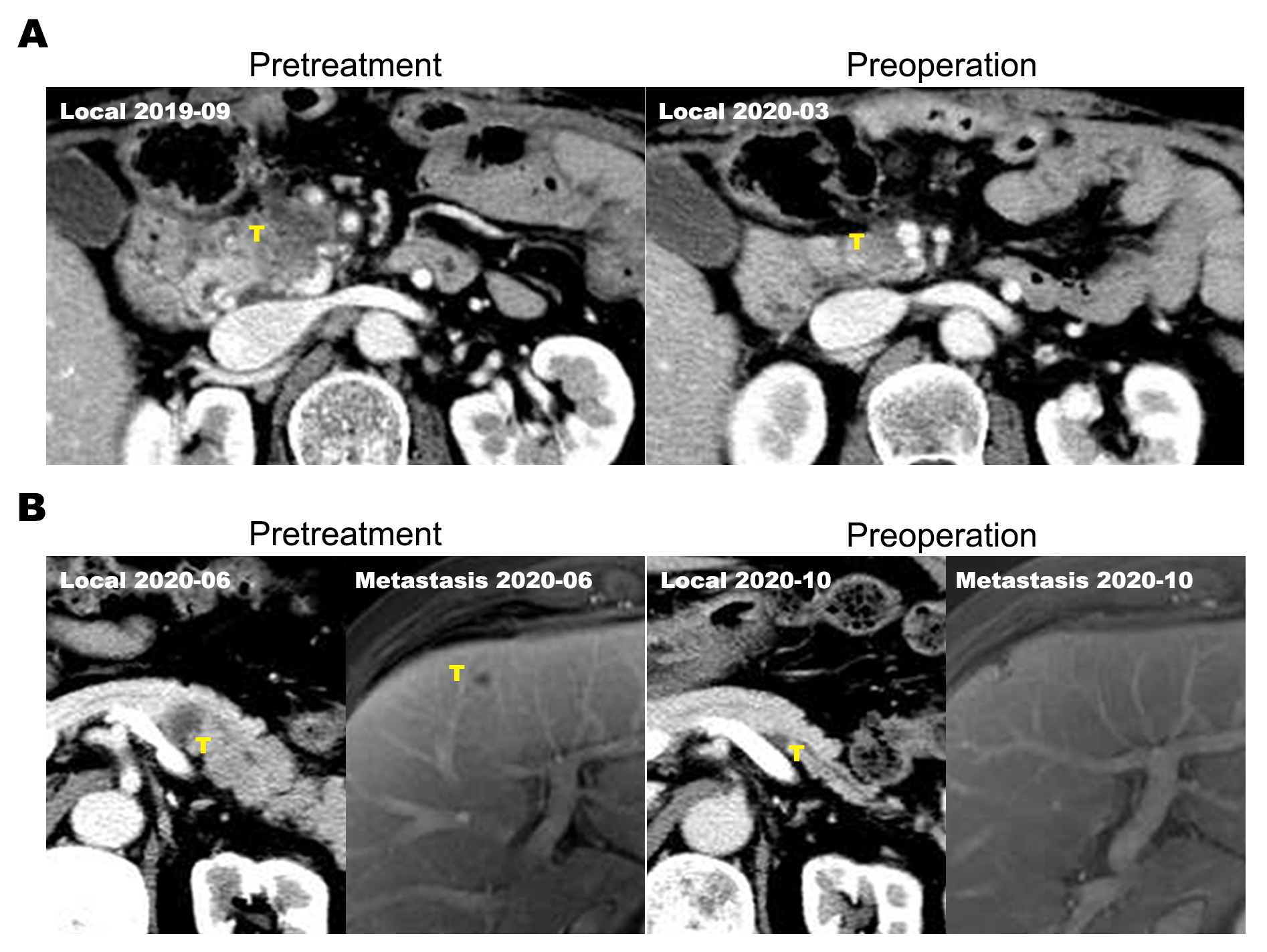

Supplement: Supplementary file 1 — Additional file 1: Supplementary Figures 1-Figure 3. [file 12885_2023_10529_MOESM1_ESM.zip › SFigure 1 The treatment response of UPC cases.TIFF]

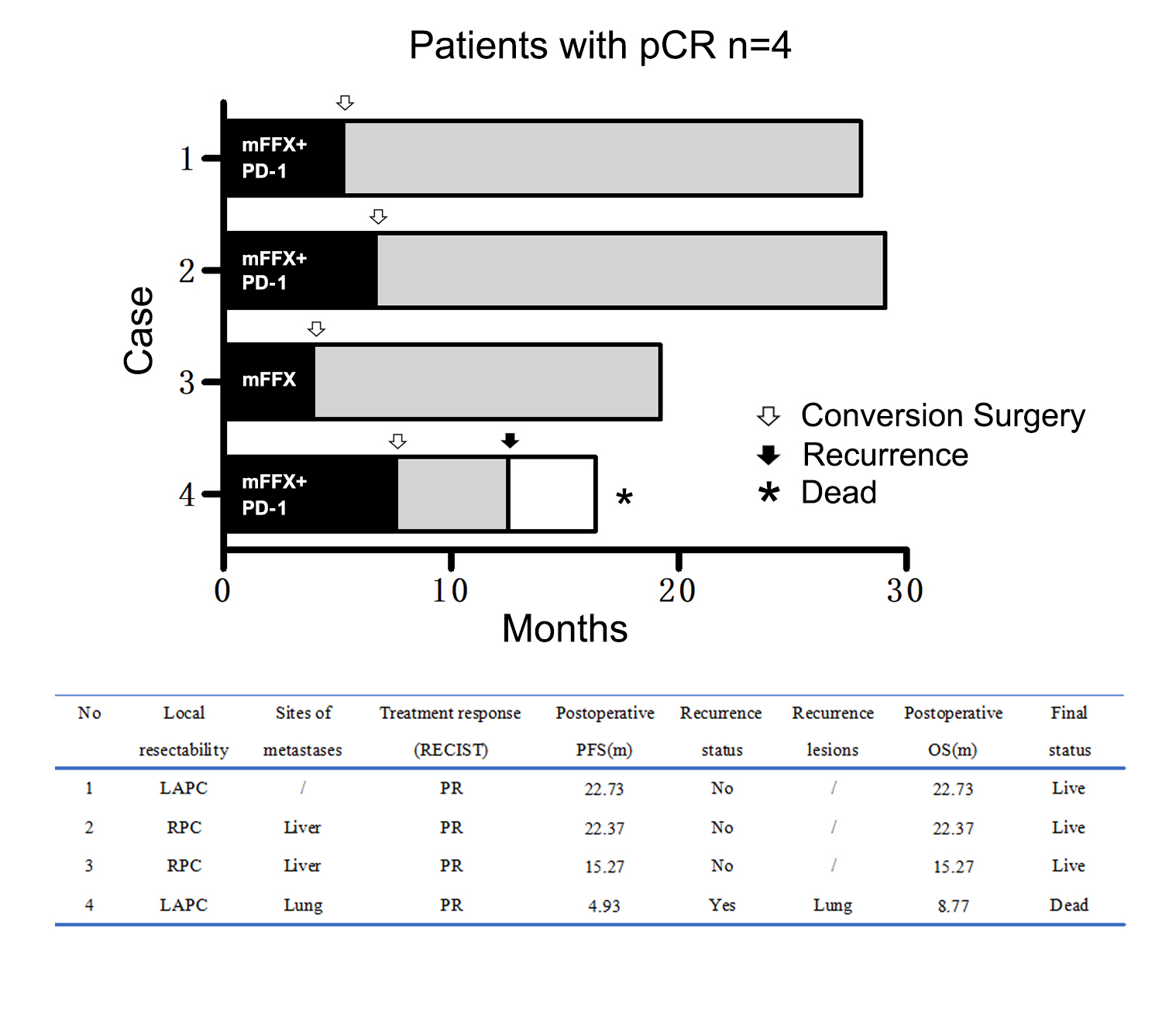

Supplement: Supplementary file 1 — Additional file 1: Supplementary Figures 1-Figure 3. [file 12885_2023_10529_MOESM1_ESM.zip › SFigure 2 The details of patients with pCR.TIFF]

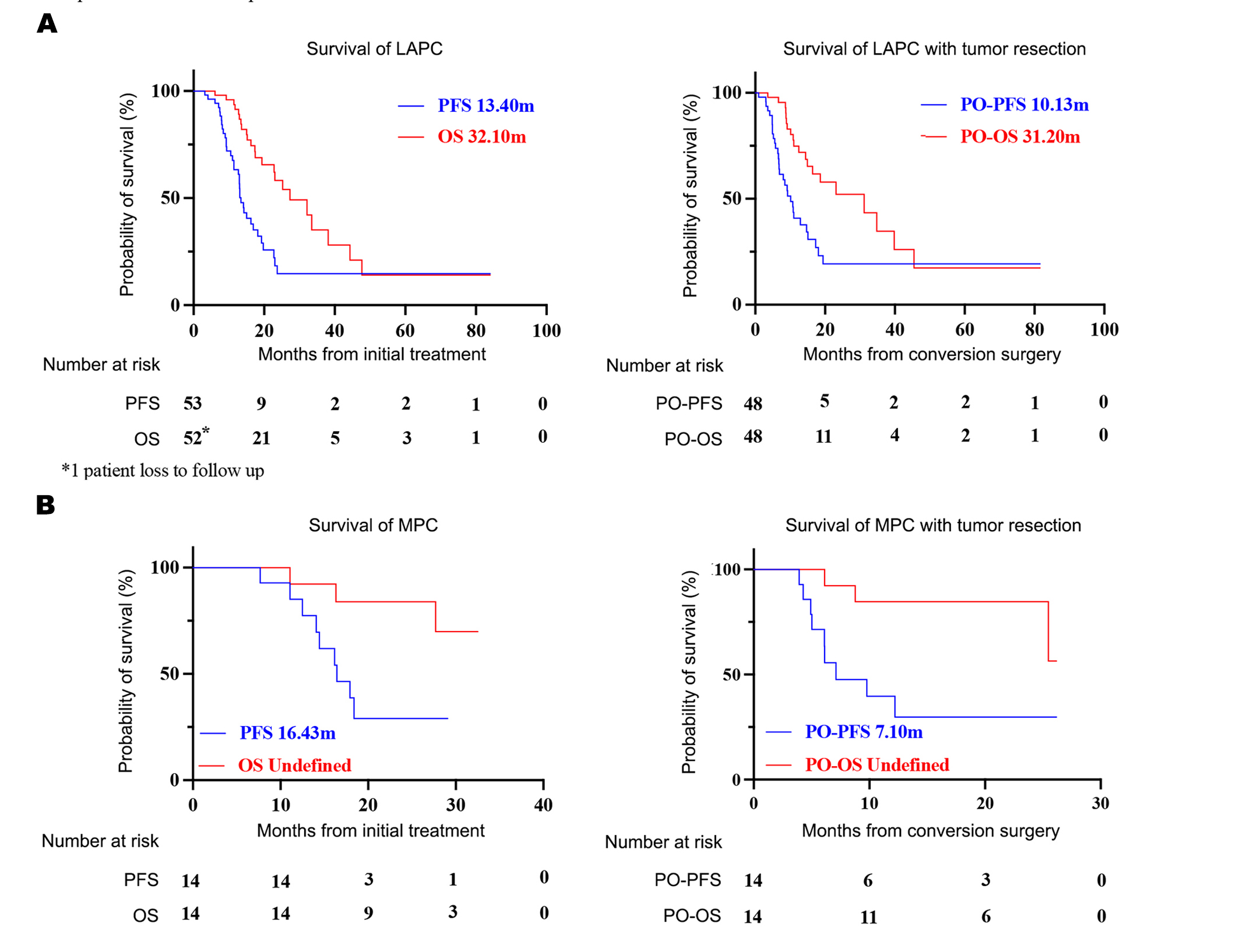

Supplement: Supplementary file 1 — Additional file 1: Supplementary Figures 1-Figure 3. [file 12885_2023_10529_MOESM1_ESM.zip › SFigure 3 The survival data of LAPC and MPC patients.TIFF]
