# Supplementary material for: Normalization of tumor markers and a clear resection margin affect progression-free survival of patients with unresectable pancreatic cancer who have undergone conversion surgery
Source: BMC Cancer. 2023 Jan 14;23:49. doi: 10.1186/s12885-023-10529-7 (PMC9840266; doi:10.1186/s12885-023-10529-7)
Supplement: Supplementary file 2 — Additional file 2: Supplementary Table 1. [file 12885_2023_10529_MOESM2_ESM.docx]

**Supplementary Table 1** Details of conversion surgery classified by resectability

|  | **LAPC**  **n = 49^a^** | **MPC**  **n = 14** | **UPC**  **n = 63^a^** |
| --- | --- | --- | --- |
| **Type of procedure** |  |  |  |
| PD | 25 (51) | 6 (43) | 31 (49) |
| DP | 13 (27) | 7 (50) | 20 (32) |
| DP-CAR | 10 (20) | 1 (7) | 11 (17) |
| TP | 1 (2) | 0 (0) | 1 (2) |
| **Duration (min)** | 326 (195–748) | 267 (133–536) | 318.5 (133–748) |
| **Estimated blood loss (mL)** | 200 (50–1300) | 150 (50–1300) | 200 (50–1300) |
| **Margin (microscopic)** |  |  |  |
| R0 | 41 (84) | 10 (93) | 51 (81) |
| R1 | 8 (16) | 0 (0) | 8 (13) |
| R2 | 0 (0) | 4 (7) | 4 (6) |
| **Treatment of metastases** |  |  |  |
| Complete response | - | 3 | 3 |
| Resection | - | 8 | 8 |
| Adjuvant therapy | - | 3 | 3 |
| **Postoperative duration of hospital stay (days)** | 14 (6–53) | 13 (5–51) | 14 (5–53) |
| **Complications** |  |  |  |
| Pleural effusion | 22 (45) | 6 (42) | 28 (44) |
| Ascites | 15 (31) | 7 (50) | 23 (36) |
| Chylus fistula | 13 (26) | 1 (7) | 14 (22) |
| Pancreatic fistula | 11 (22) | 2 (14) | 13 (21) |
| Infection | 7 (14) | 3 (21) | 10 (16) |
| **Clavien**–**Dindo grade** |  |  |  |
| 0 | 8 (16) | 3 (21) | 11 (17) |
| I | 18 (37) | 7 (50) | 25 (40) |
| II | 10 (16) | 2 (14) | 12 (19) |
| III | 12 (24) | 2 (14) | 14 (22) |
| IV | 0 (0) | 0 (0) | 0 (0) |
| V | 1 (2) | 0 (0) | 1 (2) |
| **30-day unplanned readmission to hospital** | 9 (18) | 0 (0) | 9 (14) |
| **Postoperative death (within 90 days)** | 1 (2) | 0 (0) | 1 (2) |
| **Unplanned reoperation** | 4 (8) | 1 (7) | 5 (8) |

^a^Four patients who continued to have unresectable pancreatic cancer intraoperatively were excluded

***PD*** pancreatoduodenectomy; ***DP*** distal pancreatectomy; ***TP*** total pancreatectomy; ***DP-CAR*** distal pancreatectomy with celiac axis resection*.*
