# Supplementary material for: Normalization of tumor markers and a clear resection margin affect progression-free survival of patients with unresectable pancreatic cancer who have undergone conversion surgery
Source: BMC Cancer. 2023 Jan 14;23:49. doi: 10.1186/s12885-023-10529-7 (PMC9840266; doi:10.1186/s12885-023-10529-7)
Supplement: Supplementary file 3 — Additional file 3: Supplementary Table 2. [file 12885_2023_10529_MOESM3_ESM.docx]

|  | **Postoperative PFS** | | | | **Postoperative OS** | | | |
| --- | --- | --- | --- | --- | --- | --- | --- | --- |
|  | **Univariate** **analysis** | **Multivariate analysis** | | | **Univariate analysis** | **Multivariate analysis** | | |
|  | **P** | **P** | **HR** | **95%CI** | **P** | **P** | **HR** | **95%CI** |
| **Age** (>60 *vs*. ≤60 years) | 0.724 | - | - | - | 0.840 | - | - | - |
| **Sex** (male *vs*. female) | 0.297 | - | - | - | 0.431 | - | - | - |
| **Location of pancreatic tumor** (proximal *vs*. distal) | 0.604 | - | - | - | 0.184 | - | - | - |
| **Tumor diameter** (<20 *vs*. ≥20 mm) | 0.435 | - | - | - | 0.627 | - | - | - |
| **Margin** (R0 *vs*. R1 and R2) | <0.001 | <0.001 | 0.127 | 0.042–0.386 | 0.132 | - | - | - |
| **LN metastasis** ((+) *vs*. (−)) | 0.608 | - | - | - | 0.130 | - | - | - |
| **RECIST** (PR and CR *vs*. SD) | 0.208 | - | - | - | 0.120 | - | - | - |
| **Duration of systemic treatment** | 0.218 | - | - | - | 0.066 | - | - | - |
| **TNM staging** (0, I and II *vs*. III and IV) | 0.686 | - | - | - | 0.904 | - | - | - |
| **Postoperative change in CA19-9/CEA**  (decrease to normal *vs*. not normalized) | <0.001 | 0.001 | 0.202 | 0.078–0.526 | 0.033 | 0.042 | 0.340 | 0.121-1.959 |
| **CAP grading system** (0 and 1 *vs*. 2 and 3) | 0.665 | - | - | - | 0.961 | - | - | - |

**Supplementary Table 2** Factors influencing the postoperative survival of LAPC patients
