# Supplementary material for: Normalization of tumor markers and a clear resection margin affect progression-free survival of patients with unresectable pancreatic cancer who have undergone conversion surgery
Source: BMC Cancer. 2023 Jan 14;23:49. doi: 10.1186/s12885-023-10529-7 (PMC9840266; doi:10.1186/s12885-023-10529-7)
Supplement: Supplementary file 4 — Additional file 4: Supplementary Table 3. [file 12885_2023_10529_MOESM4_ESM.docx]

|  | **Postoperative PFS** | | | | **Postoperative OS** | | | |
| --- | --- | --- | --- | --- | --- | --- | --- | --- |
|  | **Univariate** **analysis** | **Multivariate analysis** | | | **Univariate analysis** | **Multivariate analysis** | | |
|  | **P** | **P** | **HR** | **95%CI** | **P** | **P** | **HR** | **95%CI** |
| **Age** (>60 *vs*.≤60 years) | 0.167 | - | - | - | 0.677 | - | - | - |
| **Sex** (male *vs*. female) | 0.742 | - | - | - | 0.741 | - | - | - |
| **Location of pancreatic tumor** (proximal *vs*. distal) | 0.532 | - | - | - | 0.351 | - | - | - |
| **Tumor diameter** (<20 *vs*. ≥20 mm) | 0.240 | - | - | - | 0.456 | - | - | - |
| **Margin** (R0 *vs*. R1 and R2) | 0.256 | - | - | - | 0.393 | - | - | - |
| **LN metastasis** ((+) *vs*. (−)) | 0.252 | - | - | - | 0.326 | - | - | - |
| **RECIST** (PR and CR *vs*. SD) | 0.685 | - | - | - | 0.084 | - | - | - |
| **Duration of systemic treatment** | 0.237 | - | - | - | 0.704 | - | - | - |
| **TNM staging** (0, I and II *vs*. III and IV) | 0.342 | - | - | - | 0.456 | - | - | - |
| **Postoperative change in CA19-9/CEA level**  (decrease to normal *vs*. not normalized) | 0.830 | - | - | - | 0.284 | - | - | - |
| **CAP grading system** (0 and 1 *vs*. 2 and 3) | 0.263 | - | - | - | 0.390 | - | - | - |

**Supplementary Table 3** Factors influencing the postoperative survival of MPC patients
